# A VidEo-Based Intelligent Recognition and Decision System for the Phacoemulsification Cataract Surgery

**DOI:** 10.1155/2015/202934

**Published:** 2015-11-26

**Authors:** Shu Tian, Xu-Cheng Yin, Zhi-Bin Wang, Fang Zhou, Hong-Wei Hao

**Affiliations:** ^1^Department of Computer Science and Technology, School of Computer and Communication Engineering, University of Science and Technology Beijing, Beijing 100083, China; ^2^National Engineering Research Center for Information Technology in Agriculture, Beijing 100089, China; ^3^Institute of Automation, Chinese Academy of Sciences, Beijing 100190, China

## Abstract

The phacoemulsification surgery is one of the most advanced surgeries to treat cataract. However, the conventional surgeries are always with low automatic level of operation and over reliance on the ability of surgeons. Alternatively, one imaginative scene is to use video processing and pattern recognition technologies to automatically detect the cataract grade and intelligently control the release of the ultrasonic energy while operating. Unlike cataract grading in the diagnosis system with static images, complicated background, unexpected noise, and varied information are always introduced in dynamic videos of the surgery. Here we develop a Video-Based Intelligent Recognitionand Decision (VeBIRD) system, which breaks new ground by providing a generic framework for automatically tracking the operation process and classifying the cataract grade in microscope videos of the phacoemulsification cataract surgery. VeBIRD comprises a robust eye (iris) detector with randomized Hough transform to precisely locate the eye in the noise background, an effective probe tracker with Tracking-Learning-Detection to thereafter track the operation probe in the dynamic process, and an intelligent decider with discriminative learning to finally recognize the cataract grade in the complicated video. Experiments with a variety of real microscope videos of phacoemulsification verify VeBIRD's effectiveness.

## 1. Introduction

As the foremost cause of blindness, cataract, the “clouding” or opacity developed in the crystalline lens of human eyes, blinds or is weakening tens of thousands of peoples' vision all over the world [1]. It is most commonly due to aging but has many other causes such as genetics [2], trauma, drug use, radiation, and medications. The world health reports published by World Health Organization (WHO) show that the proportion of cataract patients in the global blindness increases quickly from 43% in year 1998 [3] to 47.8% in year 2002 [4], which means that about 18 million people suffer from cataract in year 2002. Based on the locations of developed opacity, age-related cataracts are categorized into three classes: posterior subcapsular cataract, cortical cataract, and nuclear cataract [5]. Among them, the nuclear cataract is one of the most common types, which is the focus in this paper.

Medically speaking, extracapsular cataract extraction is a conventional manual cataract surgery for dealing with hard cataracts; meanwhile phacoemulsification is the most widely used surgery for relatively soft cataracts. There are several typical steps of operation for the phacoemulsification surgery, for example, anesthetic, corneal incision, capsulorhexis, phacoemulsification, and irrigation and aspiration. The procedure of phacoemulsification, one key step of the surgery, can be summarized as follows [6, 7]. First, the surgeon observes the situation of the patient's cataract through microscope. Next, the grade of the opacity severity of the nuclear cataract is decided and assigned by the surgeon based on his own clinical experience. Simultaneously, a proper amount of ultrasonic energy is released with his feet. The cataract in front of the probe is gradually emulsified. Compared to the conventional extracapsular cataract extraction surgery, the phacoemulsification surgery has many advantages: small incision, short operative time, quick recovery, no need of hospitalization, small amounts of astigmatism, and fewer postoperative visits [7]. However, the phacoemulsification step is strongly dependent on the ophthalmologist's high skills and his long-term clinical experience, which nowadays is a main barrier to popularize the commonly used phacoemulsification operation instruments, especially in small and rural hospitals.

One vital factor is about the key equipment, the phacoemulsification instrument, which is not an automatically controlled machine. In some computer-assisted systems, only the images of the patient's eye are captured to the legibility for the surgeons, but the hardness level of lens nuclear and the amount of ultrasonic energy released are still manually determined by surgery operators by comparing the image with several standard pictures [8–10]. These semiautomatic systems require the surgeons to have a high skill with a rich experience to determine the hardness level and thereafter to release the right amount of ultrasonic energy. At the same time, there are also some other research efforts with several automatic grading systems established to measure the opacity severity quantitatively [11–14]. Specifically, Duncan et al. first extracted three features, that is, nuclear mean gray level, slope at the posterior point of profile, and the fractional residual of the polynomial least-square regression, and then trained a neural network classifier for determining the grade of nuclear opacity [13]. Fan et al. detected the visual axis, identified the ocular landmark features, and constructed a linear regression model for classifying the nuclear hardness [14]. Similarly, Li et al. proposed a computer-assisted method for nuclear cataract grading from slit-lamp images using ranking in the cataract diagnosis system [15, 16]. In their method, a grade indicating the severity of nuclear cataract is via ranking with some learning and prediction techniques rather not manually assigned by a trained ophthalmologist. After the grade ranking, the conventional clinical decision-making process is performed. Actually, all these automatic grading systems are based on static images, which are helpful to diagnose the cataract and recognize the cataract grade before treatment. However, as we know, the cataract grade usually varies over the procedure of the phacoemulsification surgery. Consequently, it is really challenging to intelligently track the cataract and classify the grade in the dynamic video of the operation.

Ideally, the eventual goal is to develop an intelligent system to support the phacoemulsification surgery for automatically recognizing the cataract grade and releasing the ultrasonic energy during its operation but not to simply diagnose the disease before treatment. This first step with “virtual reality” is to automatically detect the cataract location, track the operation probe, and classify the cataract in the real microscope videos of the phacoemulsification surgery. Consequently, unlike cataract grading by the opacity severity in the cataract diagnosis system with static slit-lamp images, in multimedia understanding of the phacoemulsification surgery, there are several typical challenges. First, the complicated background is always generated not only by the cataract eye but also by the surgery operation. Second, the dynamic surgery introduces complex noise with the ophthalmologist operation, for example, tissue changing, illumination varying, and probe moving. Third, the cataract grade varies over the surgery procedure. For example, the cataract grade will become lower and lower with the energy releasing on the same location. In some other cases, the cataract grade will suddenly change from one place to another when moving the probe in the surgery [6].

To our knowledge, there are very few research efforts [17] by utilizing intelligent video analysis and pattern recognition technologies to improve the automatic level of this phacoemulsification surgery. In this paper, we develop a Video-Based Intelligent Recognition and Decision (VeBIRD) system, which provides a generic framework for automatically tracking the operation process and classifying the cataract grade in microscope videos of the phacoemulsification cataract surgery. These microscope videos record the operation process in the patient's eye by the surgeon in real time. In VeBIRD, first a robust eye (iris) detector is constructed with randomized Hough transform and precisely locates the eye in the noise background. Next, an effective probe tracker is learned with Tracking-Learning-Detection (TLD) [18] and dynamically tracks the operation probe in the surgery process. Finally, an intelligent decider is trained with a discriminative learning algorithm and accurately recognizes the cataract grade in the complicated video. We make full use of the recent video analysis and pattern classification techniques to be adaptable to the phacoemulsification surgery situation. Specifically, we propose an improved randomized Hough transform method to robustly detect ellipse with noise. An adaptive TLD approach is also constructed to handle the problem that the probe is easily recognized as background. Additionally, VeBIRD has been experimented with a variety of real microscope videos of the phacoemulsification surgery, and the experimental results show that VeBIRD has the potential ability to reduce the complexity of the cataract operation and may be used to raise the automatic level of phacoemulsification instruments in the future.

## 2. Methods

### 2.1. Framework of VeBIRD

**Figure 1:**
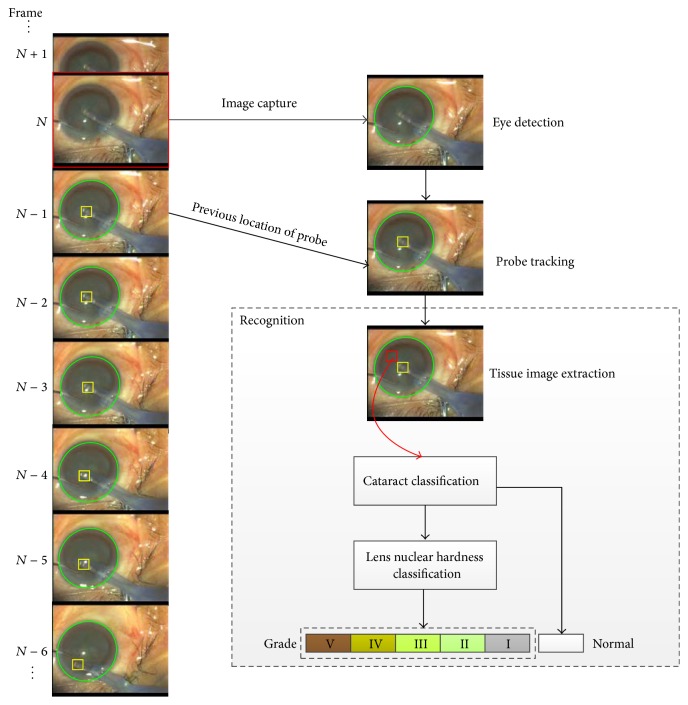
The framework VeBIRD, a Video-Based Intelligent Recognition and Decision system, for the phacoemulsification cataract surgery, where the microscope videos record the operation process in the patient's eye by the surgeon in real time. This framework mainly comprises eye detection, probe tracking, and cataract grading.

Summarily, VeBIRD uses image processing, object tracking, and pattern recognition technologies to detect the eye (eye detection), track the emulsification probe (probe tracking), and recognize the cataract grade (cataract grading). The framework of this system is shown in Figure 1, where cataract grading is composed of feature representation, cataract identification, and lens nuclear hardness classification. In Figure 1, the green ellipse represents the detected eye (iris), the yellow rectangle shows the tracked probe, and the red rectangles are the location of extracted tissues for cataract grading.

In VeBIRD, first, eye detection is performed by an eye detector which is constructed with an improved randomized Hough transform approach. Ellipse detection with this improved Hough transform is robust to noise and shape distortion. Then, a probe tracker is conducted to effectively track the emulsification probe in the eye region. Here, the tracker is built by an adaptive TLD method, which can clearly distinguish between foreground and background and therefore precisely track the object (probe) in the video. Moreover, this online tracking algorithm can be conveniently adapted and easily used in the real phacoemulsification surgery in the future. Next, an intelligent decider is used to recognize the cataract grade. In this final cataract grading step, features are extracted from the tissue image in front of the emulsification probe location; and the hardness level of the tissue (cataract grade) is predicted through a discriminative learning classifier with Support Vector Machines (SVMs) [19, 20]. Specifically, the last prediction procedure is divided into 2 substeps. The first substep is cataract identification, which identifies whether the tissue is normal or not. The other one is lens nuclear hardness classification, which classifies the hardness grade of the cataract. In the proposed system, the training databases for these classifiers are composed of images taken from real microscope videos of phacoemulsification and the grade of nuclear opacity is annotated by the experienced ophthalmologists.

Our system can be explained by the probability theory as follows. We define *I*_1:*t*_ as all the images from frame 1 to *t*, *L*_1:*t*_ as the grade of nuclear opacity from frame 1 to *t*, *D*_1:*t*_ as the results of eye detection from frame 1 to *t*, and *T*_1:*t*_ as the results of probe tracking from frame 1 to *t*. For simplicity, the subscript can be dropped. Then, our goal is to get *P*(*L*∣*I*). Based on Total Probability Theorem, we get the following equation:

(1) $$\begin{array}{c} P\left(\;L\;|\;I\;\right)=\underset{D,T}{\sum }P\left(\;L\;|\;D,T,I\;\right)P\left(\;D,T\;|\;I\;\right). \end{array}$$

Then, we assume only one result of eye detection and probe tracking is possible. We get the following equation:

(2) $$\begin{array}{c} P\left(\;L\;|\;I\;\right)=P\left(\;L\;|\;D,T,I\;\right)P\left(\;D,T\;|\;I\;\right). \end{array}$$

*P*(*D*, *T*∣*I*) can be further decomposed according to Total Probability Theorem and the assumption that only one result of eye detection is possible. Consider

(3) $$\begin{array}{c} P\left(\;D,T\;|\;I\;\right)=P\left(\;D\;|\;I\;\right)P\left(\;T\;|\;D,I\;\right). \end{array}$$

At last, we get

(4) $$\begin{array}{c} P\left(\;L\;|\;I\;\right)=P\left(\;D\;|\;I\;\right)P\left(\;T\;|\;D,I\;\right)P\left(\;L\;|\;D,T,I\;\right). \end{array}$$

The terms *P*(*D*∣*I*), *P*(*T*∣*D*, *I*), and *P*(*L*∣*D*, *T*, *I*) correspond to the eye detection, probe tracking, and the cataract grading.

### 2.2. Eye Detection with Improved Randomized Hough Transform

As we know, the Hough transform [21] is an essential method for geometric shape recognition in images and is widely used in image processing and computer vision. Meanwhile, the contour of the eye (iris) in microscope videos of phacoemulsification can approximately be regarded as a circle. Hence, the Hough transform is certainly suitable for identifying the position of the iris. The standard Hough transform finds the circle by a voting strategy which is carried out by an accumulation process in the parameter space. Generally speaking, when the dimension of the parameter space is small, the Hough transform is efficient. But the computational burden becomes heavy as the dimension of the parameter space increases. To alleviate this problem, a number of researchers have developed numerous variations of the Hough transform to decompose the high-dimension parameter space into a low-dimension one by using the geometric properties, for example, symmetry [22, 23]. In these variations, the storage space and the processing time are largely reduced. On the contrary, the real peaks in the low-dimension parameter space are more easily covered by noise than the high-dimension parameter space. The randomized Hough transform [24, 25] is a solution to alleviate the problem to some extent. However, it is still easily affected by noise in complicated images and videos. Another drawback of the Hough transform is that it may be failed for shape detection and recognition when shape distortion occurs. For example, the shape of patient's iris may vary (from a circle to an ellipse) during the surgery, and the contour of the iris changes to be similar to an ellipse sometimes. As a result, the circle detection may not recognize the shape of the iris in the dynamic situation.

In order to deal with the above challenges, we propose an improvement of the randomized Hough transform for both circle detection and ellipse detection in VeBIRD. This improved randomized Hough transform restricts the distance between the sampling points with domain priors. Moreover, we also designed a cascaded detection method combining the circle detection and ellipse detection to largely reduce the computational burden of the eye detection system.

#### 2.2.1. Improved Randomized Hough Transform

In the Cartesian coordinate system, the equation of conic curve is

(5) $$\begin{array}{c} Ax^{2}+2Bxy+Cy^{2}+2Dx+2Ey+F=0, \end{array}$$

where (*x*, *y*) represents the coordinate of a pixel in the two-dimension space (e.g., an image), *A* ~ *F* are the parameters, and |*A*| + |*B*| + |*C*| ≠ 0. The discriminant *B* ^2^ − 4*AC* can be used to classify the conic curve. If *B* ^2^ − 4*AC* < 0, the equation represents an ellipse. For an ellipse, because *B* ^2^ − 4*AC* < 0, *A* ≠ 0, (5) can be converted into

(6) $$\begin{array}{c} x^{2}+2\frac{B}{A}xy+\frac{C}{A}y^{2}+2\frac{D}{A}x+2\frac{E}{A}y+\frac{F}{A}=0. \end{array}$$

After this simple mathematic transform, the number of relevant parameters is reduced to five. Then, only five different points will determine a parameter vector (*B*/*A*, *C*/*A*, *D*/*A*, *E*/*A*, *F*/*A*)^*T*^. The randomized Hough transform randomly samples groups of points which are composed by five points from the image's edges. If a parameter vector and one group of points can satisfy (6), accumulation occurs in the parameter space. Afterwards, the peak of the parameter space is found to represent the real existing ellipse on the image. In order to reduce the consuming time and the storage size by finding peaks of the parameter space, the 5-dimension parameter space can be displaced by five 1-dimension parameter spaces, which is well suited for the image containing one real ellipse [26].

**Figure 2:**
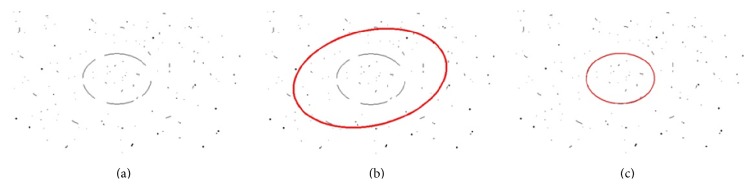
The detection results with the randomized Hough transform methods: (a) the input image with heavy noise, (b) the detection result (with red color) with the standard randomized Hough transform, and (c) the result with the adaptive randomized Hough transform.

**Figure 3:**
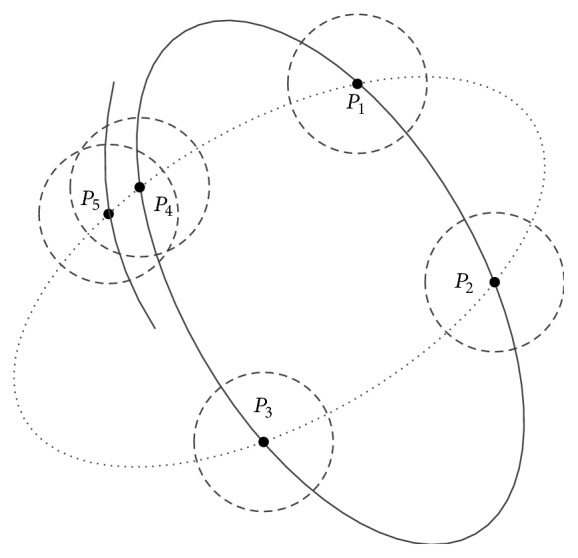
The adaptive strategy in the proposed randomized Hough transform method, where the ellipse with the full line exists while the one with the dashed line does not, and the dashed one is determined by *P*_1_ ~ *P*_5_.

In general cases where the noise of image is slight and the ellipse to be detected is relatively clear, the randomized Hough transform has a pretty good performance. However, when the image is with heavy noise (see an example shown in Figure 2), the randomized Hough transform is always failed. In the randomized Hough transform, once five noncollinear sampling points are selected randomly, the five parameters of (6) are calculated and the voting procedure is conducted in the parameter space. Actually, this aimless random sampling step introduces a plenty of invalid sampling and accumulation steps which further result in the decrease of precision and runtime in eye detection. In particular, we intuitively show this disadvantage in Figure 3. Here, the solid line ellipse represents the real existing ellipse on which the points *P*_1_ ~ *P*_4_ fall. The point *P*_5_ is a noise point which affects the ellipse detection result. If all five points are sampled together, the dashed line ellipse is detected and the wrong parameters are accumulated.

Consequently, we aim to improve this algorithm with a sampling control strategy and propose an improved randomized Hough transform approach. Unlike the aimless random sampling strategy in the conventional method, we add a constraint that each pair of sampling points must not be less than a specific threshold (shown in Figure 3 by a dashed line circle). We can observe that the error which occurred in the randomized Hough transform is avoided because point *P*_4_ and point *P*_5_ are too close to be sampled together. The distance between the real point and the noise point is often short. It is easy to get a large deviation when the parameters are calculated by a set of points which are near to each other. Because we do not sample the points which are too closer, the accuracy of eye detection is largely improved.

Please note that the runtime of circle detection is much faster than the ellipse detection. Hence, it is time-consuming to use ellipse detection all the time. To alleviate this problem, we design a cascaded detection method combining ellipse detection and circle detection. In this simple cascaded detection strategy, circle detection is first used to locate the eye (iris). If circle detection failed, ellipse detection is then utilized to further detect and locate the eye.

### 2.3. Probe Tracking with Adaptive Tracking-Learning-Detection

In the literature, there are few techniques on probe tracking for the phacoemulsification surgery. Baldas et al. proposed a simple and direct tracking algorithm [27]. They first get the eye region by thresholding the color. And then the probe is detected as a straight line in the eye region. Meanwhile, object tracking is a widely researched field in computer vision. A huge number of methods and systems of object tracking are investigated. Many effective algorithms are proposed, such as pyramidal Lucas-Kanade (LK) tracker [28], particle filter [29], and tracking-by-detection [30]. Specifically, Tracking-Learning-Detection (TLD) method [18] is the recent highlighted one. In VeBIRD, we design an adaptive TLD method to locate and track the probe by combing a priori knowledge to adaptively distinguish between foreground and background.

#### 2.3.1. Tracking-Learning-Detection

In the first frame, the real location of probe is annotated manually. Afterwards the probe is correspondingly tracked. There are 3 components in the framework of Tracking-Learning-Detection, that is,* Tracker*,* Detector*, and* Learning*. The task of* Tracker* is to estimate the object's motion between the consecutive frames and predict the location of object in the next frame.* Detector* is used to detect object in one frame without any information of other frames.* Learning* component estimates error of* Tracker* and* Detector* and generates training data for* Detector*. The final output of TLD is a combination of the results of* Tracker* and* Detector*.

*Tracker* in the TLD framework is the Median Flow tracker [31]. The object's location is estimated by a novel measure, Forward-Backward error. The object is represented by a bounding box in which a number of points are generated randomly. First, the location of the points in the next frame is predicted by a pyramidal Lucas-Kanande (LK) tracker [28]. Then the locations of the points in the current frame are predicted backward based on the predicted locations in the next frame by a pyramidal LK tracker too. At last, the distances of pairs in these two sets of points are compared, and the points are considered incorrect if they differ significantly. The bounding box enclosing all correct points is the final output of the tracker.

*Detector* in the TLD framework scans the input image by a sliding window. Then a cascaded classifier is trained to decide whether the image patches contain the object. In the first stage, the patch variance is computed and analyzed. All patches, for which the gray-value variance is smaller than half of the variance of the patch that was selected for tracking, are rejected. Next, in the second stage, left patches are to be further classified by an ensemble classifier. The feature is a binary code computed by comparing pairs of pixels which are generated offline randomly and stay fixed in runtime. Each base classifier is based on each feature independently. The output of the base classifier is the posterior probability which is gotten from the training data. The label output by the ensemble classifier is judged by whether the sum of all posterior probabilities is more than a given threshold. The last stage of the cascade is a nearest neighbor classifier for reconfirmation.

*Learning* component in the TLD framework is a P-N learning algorithm [32]. Using the conservative similarity and temporal and spatial information, the reliability of the object's location is decided. Then based on the object's location with very high confidence, the samples classified incorrectly by the detector are utilized to update the object model and the ensemble classifier.

#### 2.3.2. Adaptive Tracking-Learning-Detection

In VeBIRD, we improve the TLD method to deal with the specificity of probe tracking. In many object tracking algorithms, and also in the TLD method, an object is always modeled as a rectangle which is not the shape of the probe. As a result, a large region of the background is contained in the rectangle. The main effect for TLD is that the ensemble classifier often outputs the wrong label especially when the probe is still in the rectangle but the background is with a small change. This error easily leads TLD to regard the object as absent while tracking.

Here, we construct an adaptive Tracking-Learning-Detection (TLD) approach, where the idea is to force the ensemble classifier to output the positive label easily. The second stage of the cascaded detector is the key as the first stage is to eliminate the incredible samples and the last stage of cascade is to verify. The second stage is an ensemble classifier. Each base classifier is based on each feature independently. The output of a base classifier is the posterior probability. But the samples are gotten from the* Learning* component; hence several features are not covered in the long term of tracking. The corresponding base classifier will output 0 for a long time. To avoid the decrease of the posterior probability, we empirically select one-fifth of base classifiers randomly to set their initial posterior probability to 0.5. Then the ratio of number of positive samples and total number of samples gotten from the P-N learning algorithm is updated to the posterior probability of these base classifiers frame by frame. This simple and adaptive improvement avoids the wrong absence by considering the overall conditions of training data.

### 2.4. Cataract Grading with Support Vector Machines

After eye detection and probe tracking, the following task is cataract grading. Here, tissue image at the top of the emulsification probe is extracted and feature representation is constructed. Next, an intelligent decider with SVMs first identifies whether the tissue is normal or not and then classifies the hardness degree (grade) of the patient's cataract. Actually, the cataract grade decides the release lever of ultrasonic energy in the phacoemulsification surgery.

#### 2.4.1. Tissue Image Extraction and Feature Representation

In VeBIRD, the cataract grading system must be accurate and in real time to recognize the tissue image and classify the hardness grade at the top of the emulsification probe. In general, tissue image extraction and feature representation of the tissue image are the fundamental step.

In cataract grading, VeBIRD extracts features with color characteristics of the images, since the color is the key information for classifying the grade of the lens nuclear hardness by surgery operators. The original RGB values of image pixels are usually used to construct features. However, in real situations, they are always with varied noise in the image. Hence, we use the following strategy to extract the feature vector of the tissue image. First, the extracted tissue image part is normalized to 32 × 32. Then, we further uniformly divide the image into 8 × 8 grids. Next, we calculate the average color value of each grid as one feature. Finally, the color feature vector is computed as follows:

(7) fI=r1,g1,b1,…,rn,gn,bn,r¯1,g¯1,b¯1,…,r¯m,g¯m,b¯m,

where *I* is an image, (*r*_*i*_, *g*_*i*_, *b*_*i*_) is the color value of *i*th pixel of *I*, *n* is the total number of pixels, $\left({\bar{r}}_{j},{\bar{g}}_{j},{\bar{b}}_{j}\right)$ is the average color value of the *j*th grid, and *m* is the total number of grids.

#### 2.4.2. Identification and Classification with Support Vector Machines

According to the Emery-Little classification in phacoemulsification, the grade standard is shown in Table 1. The color features (feature representation) with some examples are also shown in Figure 7. We follow the principal criteria and categorize all images into six types. As shown in Figure 1, the cataract grading stage includes two recognition substeps, that is, cataract identification and lens nuclear hardness classification. Here, identification decides whether the tissue is normal or not, and classification determines the hardness grade of the cataract. Here we introduce the cataract identification function for avoiding the hurt of the normal tissue in the operation (in the future applications). Moreover, the lens nuclear hardness classification function can be used to control the release of ultrasonic energy while operating in the phacoemulsification surgery. Obviously, a cascaded classifier is suitable to fulfill the classification task. That is, if a tissue image is classified into the normal tissue (by cataract identification), the second classifier (grade classification) will not be applied. Otherwise, the grade of hardness is recognized by the second stage of the cascaded classifier. Such a strategy can not only reduce the computation complexity but also avoid hurting the normal tissue. Specifically, the designed classifiers for both identification and classification are SVMs with a RBF (Radial Basis Function) kernel. As described before, all the training and testing images are normalized to the same size to ensure the same dimension of extracted features (see (7)).

## 3. Results

In this section, we will sequentially describe the experimental datasets and experiments of eye detection, probe tracking, and cataract grading techniques in VeBIRD. Our eye detection method is compared with the randomized Hough transform method. Our probe tracking method is compared with TLD. At last, we compare our cataract grading method with *K*NN.

### 3.1. Implementation and Data Subsets for Evaluation

Our system has been implemented in VC++ using the OpenCV library on a 2.6 GHz Quad-Core Intel Core i5 CPU with 8 GB RAM. We use a variety of real videos of the phacoemulsification surgery in the patient's eye acquired with the microscope in one month, which are captured from the operation procedure of the real phacoemulsification surgery from Beijing Tongren Hospital of China [6]. The research on the data has been approved by the ethics committee of Beijing Tongren Hospital. All data have been anonymized and deidentified.

We perform 3 types of experiments, that is, experiments with eye detection, experiments with probe tracking, and experiments with cataract grading. Hence 3 different subsets of microscope videos/frames are constructed and annotated, respectively. Specifically, for experiments with eye detection, the focus is to evaluate the precision of the eye detection. So, we randomly sample 2000 video frames of all these videos and then perform the proposed eye detection method on this dataset. For experiments with probe tracking, 5 typical videos with more than 100,000 frame are used to evaluate the tracking performance quantitatively. For experiments with cataract grading, the identification/classification accuracy is our target. Similarly, 2000 video frames are randomly selected at proper intervals from videos and the hardness level of cataract for each frame is annotated by the experts (skilled ophthalmologists). All datasets are divided into the training and testing sets. The detailed information about them are shown in Table 2.

### 3.2. Experimental Results with Eye Detection

**Figure 4:**
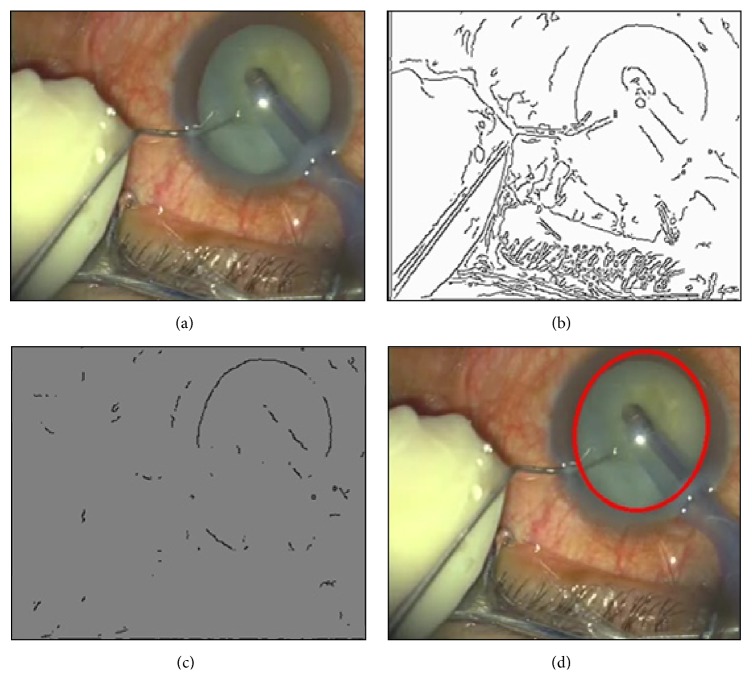
The (example) eye detection procedure in VeBIRD: (a) the original image of the cataract surgery, (b) the result with edge detection, (c) the denoised result, and (d) the final result with the improved randomized Hough transform approach.

In VeBIRD, the detected region of the eye (iris) is to constrain the location of the probe, and afterwards the abnormal tissue in front of the probe is extracted for analysis. Obviously, the detection error will be accumulated to the next step. One example of the eye detection procedure is shown in Figure 4.

**Figure 5:**
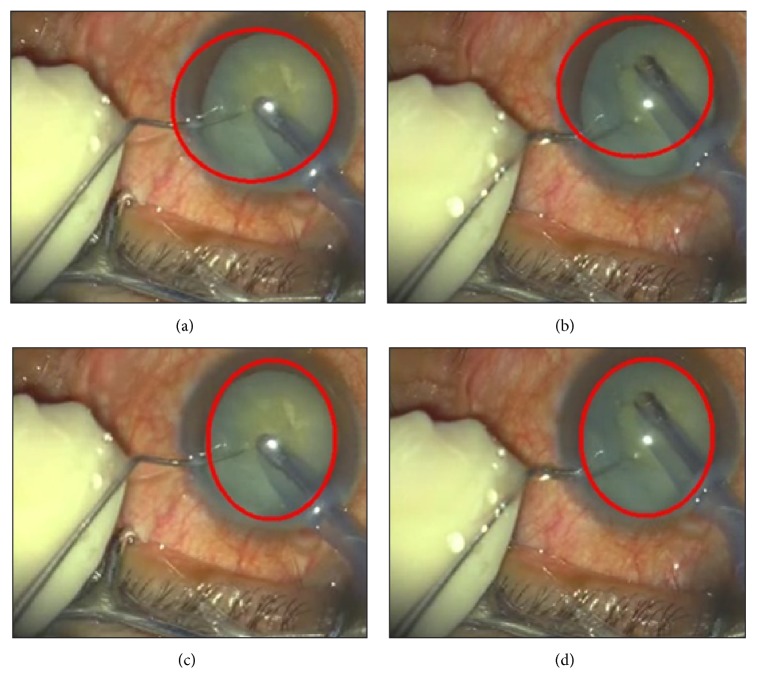
Comparison results between the standard randomized Hough transform method and the improved one: (a) and (b) are the results from the standard method, while (c) and (d) are the corresponding results detected by the improved method.

First, we show some qualitative comparison results of the randomized Hough transform method and the improved approach (see Figure 5). From Figure 5, we can see that when the shape of iris varies, the proposed method is much better than the standard randomized Hough transform. Specifically, if the iris is represented by a circle, some parts of the normal tissue are likely in the circle and other parts are easily outside the circle. Moreover, the whole iris is located by the proposed approach since the improved standard randomized Hough transform technique can adaptively detect shapes varying from circle to ellipse.

Second, we also present a quantitative performance. As the contour of the iris is not a standard circle or a standard ellipse, the error of the detected results is difficult to quantify. Here, we propose a simple specific-domain strategy to evaluate the detection result. That is, if the detected ellipse encircles the iris, and at the same time it is encircled by the spatia anguli iridocornealis (the line separating the iris and the “white eyeball”), then the result is regarded as a correct one. Consequently, the detection accuracy of the proposed technique is described in Table 3 on the testing set. In most cases, this method can correctly detect the whole iris (with 92% accuracy). Table 3 also shows that the detection performance can be affected by the number of sampling points in the Hough transform algorithm. Generally speaking, the larger number of sampling points the better performance.

### 3.3. Experimental Results with Probe Tracking

We use a quantitative metric to evaluate the tracking algorithm, namely, precision (*P*). Define the overlapping rate (OR) as

(8) $$\begin{array}{c} \text{OR}=\frac{\text{area}\left(ROI_{T}\cap ROI_{G}\right)}{\min\left\{\text{area}\left({ROI}_{T}\right),\text{area}\left(ROI_{G}\right)\right\}}, \end{array}$$

where *ROI*_*T*_ is the result of the tracker and *ROI*_*G*_ is the ground truth. The tracking result is considered as a correct one when OR is larger than a specific threshold. In VeBIRD, we set the threshold as 0.5. Note that because we cannot annotate the ground truth exactly since the scale of the probe is defined as unclear the metric “OR” is defined with a little relaxation compared to the conventional one. Here, the precision *P* is calculated as

(9) $$\begin{array}{c} P=\frac{{\#}_{\text{correct}}}{{\#}_{\text{frames}}}, \end{array}$$

where #_right_ is the number of frames in which the tracking result is correct and #_frames_ is the total number of all frames in the video.

**Figure 6:**
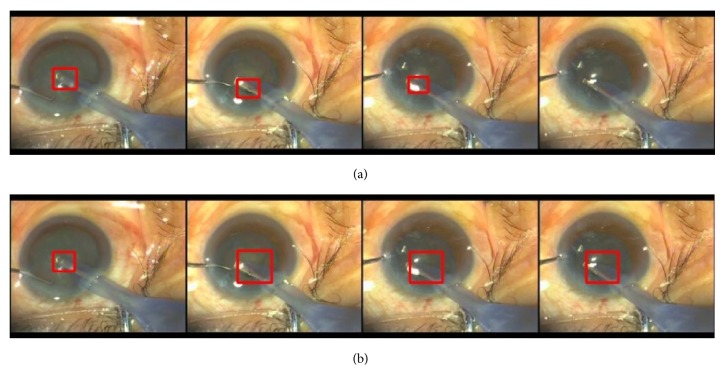
Probe tracking example results in VeBIRD: (a) and (b) are the results from Tracking-Learning-Detection (TLD) and the adaptive TLD methods, respectively. The two rows (from left to right) show the tracking results in frames 1130, 1207, 1222, and 1223 of Video 1.

**Figure 7:**
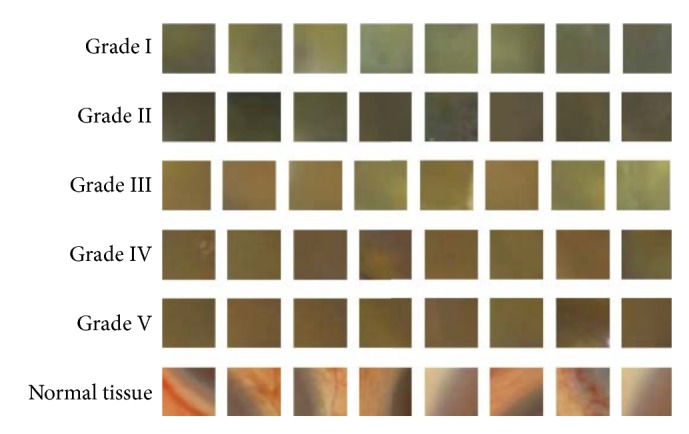
Feature representation with different color information for cataract grades.

The quantitative results on the testing video set of TLD and the adaptive TLD methods are compared in Table 4. We can see that the improvement of our method is pretty large by increasing the precision of more than 20%. The adaptive TLD method obtains a better performance than TLD for all videos. Specifically, for Video 3, the object is lost by TLD and never recovered. But our adaptive TLD can effectively track the probe accurately. We also show some typical different results between the original TLD and the adaptive TLD methods in Figure 6. Figure 6 shows that the biggest problem of TLD is that it tends to classify some of the images of the probe as negative samples. For example, the difference of the background's appearance and the probe's one is small between frame 1222 and frame 1223. The tracker of TLD is disabled to track the probe and outputs a wrong negative label, which results in the absence of the tracking result (the red rectangle in Figure 6). In the adaptive TLD method, though the tracker fails to track the probe, the detector works well. Hence, the probe region is correspondingly retrieved. Moreover, the detector is updated by more correct samples to be more accurate in the long term of probe tracking in VeBIRD.

### 3.4. Experimental Results with Cataract Grading

In experiments with cataract grading, there are two tasks, that is, cataract identification and hardness classification, which are performed and analyzed with numerous video frames. In the experiment of hardness classification, we compare two types of classifiers for identification and classification, that is, *K* Nearest Neighbor Classifiers (*K*NN) and Support Vector Machines (SVMs), where SVM is a recent representative discriminative approach in pattern recognition and machine learning fields. Moreover, the *K*NN classifier is with 5 neighbors, and the parameters of SVMs with RBF kernel are decided with cross-validation by LIBSVM [19]. However, in the experiment of cataract identification, we only use the SVM classifiers as it is a simple two-class recognition task.

The experimental results with the average recognition rate are shown in Table 5, which show that the identification and classification techniques have an impressive performance. First, the identification accuracy is very high, that is, 99.2%, because of the simple task. Second, classification accuracies (recognition rates) of both classification methods (*K*NN and SVM) are more than 90%. SVM has a much better performance with 96.3%. Consequently, the SVM classifier is finally selected as lens nuclear hardness classification in VeBIRD. We also analyze the failed cases for hardness classification. As we know, the successive and adjacent grades (e.g., 2 and 3 or 3 and 4) are easy to be confusedly classified by surgeons, even skilled experts. Hence, this observation also can explain most of the failed cases in the experiments. Namely, in some times, the hardness classification decider will classify a very few successive and adjacent grades in video frames.

## 4. Conclusion

In this paper, we introduce design principles, key technologies, and experimental results of VeBIRD (a Video-Based Intelligent Recognition and Decision system) for the phacoemulsification cataract surgery. By utilizing a variety of video analysis and pattern recognition techniques, this new technology can intelligently analyze real videos from the operation process; that is, it robustly detects the eye (iris), effectively tracks the emulsification probe, and precisely grades the hardness of lens nuclear. Specifically, we highlight three major contributions of VeBIRD. First, VeBIRD provides a generic framework (with eye detection, probe tracking, and cataract grading) for tracking the operation process and classifying the cataract grade in microscope videos of the phacoemulsification surgery. Second, several novel techniques are designed in VeBIRD. For example, the proposed randomized Hough transform is robust to noise and shape distortion. The adaptive TLD method for object (probe) tracking can easily distinguish between foreground and background. Third, a variety of experiments on real microscope videos of the phacoemulsification surgery verify that VeBIRD can automatically and effectively detect the eye, track the probe, identify the intraocular tissue, and classify the hardness of lens nucleus.

Imagine that, in the computer-aided system with intelligent programs of the real phacoemulsification surgery in the future, the cataract grade (the hardness of lens nucleus) is automatically decided, and correspondingly the release of the ultrasonic energy is also automatically controlled. In such cases, the surgeons only need to simply move and control the probe. Obviously, VeBIRD is one potential technology for these intelligent computer programs. Actually, only 2 steps need to be changed or added in VeBIRD. First, the images/frames are not from the prestorage videos but from the real-time captured ones with the phacoemulsification instrument. Second, one controlling module should be added to decide the release of proper ultrasonic energy according to the hardness degree recognized by cataract grading and afterwards to emulsify the cataract. With the help of the computer-aided system, the difficulty level of the cataract operation and its heavy reliance on highly skilled surgeons should be significantly reduced. Hence, it will help promote the spread of the surgery, which will be possible for operating in not only big and advanced hospitals but also small and rural ones. Of course, the proposed system could still be improved in some aspects. First, the hardness of the nucleus depends not only on color but also on some other factors such as shape and scale. We will collect and annotate more data to improve the system. Second, the amounts of ultrasound use are mainly decided by the hardness of the nucleus, but they are not linearly dependent. We will mine their relation in the next step.

We hope that continuous efforts will help improve the automatic level of phacoemulsification instruments and intelligentize the phacoemulsification surgery which can be really operated by general surgeons in the future.

## Figures and Tables

**Table 1:** Grades of the lens nuclear hardness with the Emery-Little classification strategy in phacoemulsification and feature representation (color information) of the tissue image.

Classification	Medicine name	Lens nuclear color	Label
Normal tissue	—	—	0
Grade I	Very soft nuclear	Transparent and nonnuclear	1
Grade II	Soft nuclear	Yellow or yellow-white	2
Grade III	Medium hard nuclear	Dark yellow	3
Grade IV	Hard nuclear	Brown or amber	4
Grade V	Extremely hard nuclear	Dark brown or black	5

**Table 2:** The training and testing sets in the experiments for eye detection, probe tracking, and cataract grading.

	Training set	Testing set
Eye detection (video frames)		
	1000	1000

Probe tracking (videos)		
	Video 1	Videos 2, 3, 4, and 5

Cataract grading (video frames)		
Normal tissue	446	444
Grade I	293	296
Grade II	184	183
Grade III	37	33
Grade IV	28	30
Grade V	12	14

Total	1000	1000

**Table 3:** The accuracy of eye detection with different numbers of sampling points in video frames on the testing set.

Sampling number	3000	5000	8000	10000

Accuracy (%)	77.2	84.1	89.4	92.3

**Table 4:** The performance of probe tracking on the testing set of the standard Tacking-Learning-Detection (TLD) and the adaptive TLD methods.

Video	Precision of adaptive TLD (%)	Precision of TLD (%)
2	84.5	61.2
3	91.4	15.5
4	98.2	82.0
5	76.2	64.4

**Table 5:** The recognition rates of cataract grading (including cataract identification and hardness classification) with *K* Nearest Neighbor Classifiers (*K*NN) and Support Vector Machines (SVM).

	Identification	Classification
*K*NN	—	92.5%
SVM	99.2%	96.3%
